# The evidence base for psychotropic drugs approved by the European Medicines Agency: a meta-assessment of all European Public Assessment Reports

**DOI:** 10.1017/S2045796020000359

**Published:** 2020-04-27

**Authors:** Florian Erhel, Alexandre Scanff, Florian Naudet

**Affiliations:** Clinical Investigation Center (INSERM 1414), Rennes University Hospital, Rennes, France

**Keywords:** Psychopharmacology, psychotropic drugs, randomised controlled trials, research design and methods, systematic reviews

## Abstract

**Aims:**

To systematically assess the level of evidence for psychotropic drugs approved by the European Medicines Agency (EMA).

**Methods:**

Cross-sectional analysis of all European Public Assessment Reports (EPARs) and meta-analyses of the many studies reported in these EPARs. Eligible EPARs were identified from the EMA's website and individual study reports were requested from the Agency when necessary. All marketing authorisation applications (defined by the drug, the route of administration and given indications) for psychotropic medications for adults (including drugs used in psychiatry and addictology) were considered. EPARs solely based on bioequivalence studies were excluded. Our primary outcome measure was the presence of robust evidence of comparative effectiveness, defined as at least two ‘positive’ superiority studies against an active comparator. Various other features of the approvals were assessed, such as evidence of non-inferiority *v*. active comparator and superiority *v*. placebo. For studies with available data, effect sizes were computed and pooled using a random effect meta-analysis for each dose of each drug in each indication.

**Results:**

Twenty-seven marketing authorisations were identified. For one, comparative effectiveness was explicitly considered as not needed in the EPAR. Of those remaining, 21/26 (81%) did not provide any evidence of superiority against an active comparator, 2/26 (8%) were based on at least two trials showing superiority against active comparator and three (11%) were based on one positive trial; 1/26 provided evidence for two positive non-inferiority analyses *v*. active comparator and seven (26%) provided evidence for one. In total, 20/27 (74%) evaluations reported evidence of superiority *v*. placebo with two or more trials. Among the meta-analyses of initiation studies against active comparator (57 available comparisons), the median effect size was 0.051 (range −0.503; 0.318). Twenty approved evaluations (74%) reported evidence of superiority *v*. placebo on the basis of two or more initiation trials and seven based on a single trial. Among meta-analyses of initiation studies against placebo (125 available comparisons), the median effect size was −0.283 (range −0.820; 0.091). Importantly, among the 89 study reports requested on the EMA website, only 19 were made available 1 year after our requests.

**Conclusions:**

The evidence for psychiatric drug approved by the EMA was in general poor. Small to modest effects *v*. placebo were considered sufficient in indications where an earlier drug exists. Data retrieval was incomplete after 1 year despite EMA's commitment to transparency. Improvements are needed.

## Introduction

Since early 1995, European Union authorisations for many new medicinal products has been obtained through a centralised procedure. This procedure, managed by the European Medicines Agency (EMA), is mandatory for products derived from biotechnology and other high-technology procedures, those aimed at the treatment of human immunodeficiency virus/acquired immunodeficiency syndrome, cancer, diabetes, neurodegenerative diseases, autoimmune disorders, viral diseases and also orphan medicines used to treat rare diseases. The centralised authorisation application also can be submitted whenever the medicinal product involved is a major therapeutic, scientific or technical innovation, or is relevant in any other way for population health. In this context the EMA assesses the evidence presented by drug companies requesting marketing authorisations, and judges whether the known (and possibly unknown) adverse effects of a new drug are acceptable when set against the expected benefits, demonstrated in *ad hoc* randomised controlled trials (RCTs) (Bighelli and Barbui, 2012). This type of evaluation involves a complex interplay of clinical practice, pharmacology, epidemiology, policy and politics (Avorn, 2012) and comes under pressure from major conflicting interests between companies, patients, doctors and public health advocates who have sometimes very divergent opinions. Therefore, regulatory agencies' decisions are scrutinised and are often criticised, especially when based on weak evidence. In this instance, care that is offered to patients is liable to be questioned and discredited, with the risk of decreasing public trust in medicine.

Psychotropic medication is a perfect example. While these drugs have gone through strict regulatory controls, their risk–benefit balance is still the subject of heated debate (Gotzsche *et al*., 2015). Specifically, new drugs are often criticised (1) for being no more or even less effective than more long-established drugs, (2) because of questionable differences *v*. placebo in terms of clinical relevance, (3) sometimes because of specific safety concerns and (4) because of higher cost. Some argue that these concerns are merely a manifestation of the recurrent attacks on psychiatry (Nutt *et al*., 2014). On the other hand, many well-described examples, such as the approvals of nalmefene (Naudet *et al*., 2016) and, more recently, paliperidone in 3-monthly injections (Ostuzzi *et al*., 2017), suggest that the EMA's thresholds are too lenient, especially for comparative effectiveness (Garattini and Bertele, 2005).

Interestingly, the EMA has been committed for many years to promoting transparency about drug efficacy and safety data (EMA, 2018*b*). The EMA publishes a European Public Assessment Report (EPAR) for each marketing authorisation application it grants. An EPAR presents the evidence considered, describes the discussion of the Committee for Medicinal Products for Human use (CHMP), and the final recommendation of the CHMP. While the exhaustiveness of the EPARs has been criticised for not providing enough detailed information for the purpose of meta-analyses (Barbui *et al*., 2011), these documents describe all arguments retained for approving the drug. On the basis of these EPARs, we aimed to systematically assess the level of evidence on which drug approvals in psychiatry are based.

## Methods

A standard protocol was developed and registered before the beginning of the study in the PROSPERO database (systematic review registration – PROSPERO 2017:CRD42017059930).

### Eligibility criteria

We surveyed all available EPARs concerning all classes of approved psychotropic medication (drugs used in both psychiatry and addictology) for adults. Since some EPARs presented various evaluations of different applications, we focused on the different marketing authorisation applications defined by the drug, the route of administration and the indication. All years were considered. We excluded EPARs solely based on bioequivalence studies, because we were only interested in clinical evidence.

### Search strategy and selection process

Eligible EPARs were identified by two independent reviewers (Florian Erhel and Florian Naudet) from the European Medicines Agency website (http://www.ema.europa.eu/ema/) using the following path: Find medicine→Human medicines→European public assessment reports→Browse by therapeutic area→Psychiatry and Psychology. All disagreements between the two reviewers were resolved by consensus.

### Data collection

Two independent reviewers (Florian Erhel and Alexandre Scanff) collected data from all the selected EPARs using a data extraction sheet that was pilot-tested on two EPARS. For each EPAR, information was extracted on (1) characteristics of the EPAR (year, drug, route of administration, disease, indication and manufacturer of the drug) and (2) all outcomes measured as specified below.

In addition, the two reviewers collected information on all the RCTs included in these EPARs. Studies were retrieved and extracted exclusively from the EPARs (no search of the literature was performed in parallel). When there was missing information concerning these RCTs, one reviewer (Florian Erhel) contacted the EMA to obtain the corresponding study reports, which were also assessed by the two independent reviewers.

All disagreements were first resolved by consensus and then by consulting a third reviewer for arbitration (Florian Naudet).

### Outcome measures

#### Description of the EPARs

Our primary outcome measure was the presence of robust evidence of comparative effectiveness defined as at least two ‘positive’ superiority studies in an indication where an active comparator was already approved. The criterion of two positive studies was retained since it is used to establish effectiveness in the Food and Drug Administration's Guidance for Industry (FDA, 1998). In addition, we noted whether the evaluation reported (1) evidence from only one positive study (alone, or associated with one or more negative studies) or (2) no evidence at all (only negative studies, no comparison reported, or no study at all) or (3) no requirement for evidence of comparative effectiveness.

We used on the same method to describe the presence of evidence of non-inferiority against active comparator.

We described the primary outcomes used in all the EPARs demonstrating comparative effectiveness, and detailed whether these EPARs investigated a list of possible outcomes (symptom severity scale, clinical global impression, global functioning, quality of life, relapse, response, remission or any other outcome).

As secondary outcomes, we adopted the same method to describe (1) evidence of efficacy against active comparator in continuation trials (defined as studies among patients who have responded to initial treatment with the same drug and route), (2) evidence of efficacy against placebo in superiority trials and (3) evidence of efficacy *v*. placebo in continuation trials.

In addition, we reported whether the definition of the target population in the approval was based on a subgroup analysis (with a difference between pre-specified and *a posteriori* subgroup analyses). Approvals where subgroup analyses were presented but without any impact on the target population definition were considered as not based on subgroup analyses.

We checked whether the EMA reported an assessment of the risk of bias concerning the trials considered within the EPAR (and if it did, whether it was with or without a systematic assessment). If an assessment was provided, we noted whether any bias was identified.

Concerning the description of possible safety issues, we described (1) the number of patients exposed to the drug before submission for market approval and (2) the presence or absence of a reported comparison with active comparator and with placebo (yes/no/only qualitative) and the explicit mention of a safety issue in the report (yes/possible/no).

Concerning the description of possible tolerance issues, we described the rates of discontinuation due to adverse events, the presence or absence of a comparison with active comparator and with placebo (yes/no/only qualitative) and the mention of a tolerance issue (yes/possible/no).

We described the number of approvals granted but accompanied by the requirement to conduct specific post-marketing studies (with the reasons), the need for further opinion (e.g. scientific advisory group) and the possible expression of divergent positions within the CHMP in the EMA.

#### Description of the studies included in different EPARs

For each EPAR, we noted the number of studies. For each study listed in the efficacy chapter, we extracted information on study duration, design ((1) superiority/non-inferiority with non-inferiority boundaries, (2) initial, continuation), comparator(s), number of arms, number of participants per arm, effect sizes measured for the primary outcomes, type of primary outcome, numbers of withdrawals, numbers of discontinuation due to adverse events, inclusion of suicidal participants. In the case of multiple-arm studies, every arm was extracted.

### Strategy for data synthesis

A descriptive analysis was performed. It consisted of estimates for the different outcomes (numbers and percentages for categorical outcomes and means, standard error or medians and interquartile intervals for quantitative outcomes).

In addition to the descriptive analysis, effect sizes (Cohen's *d*) and their corresponding *p* values were plotted against one other for different outcomes (and for each study design) both at the study level and at the approval level for each dose of each drug (i.e. pooled effect size obtained with a random effect meta-analysis). The formulas applied to calculate the effect sizes are provided in the e-methods. Although not always intuitive, and oversimplified, it is admitted that a Cohen's *d* value of 0.2 is small, 0.5 is medium and 0.8 is large (Cohen, 2013). All analyses were performed using R language and the packages tidyverse, compute.es and meta.

## Results

On 22nd March 2017, we had identified 25 EPARs corresponding to 29 marketing authorisation applications. Two of them resulted in a refusal: agomelatine (orally) for major depressive disorder and asenapine (sublingual) for schizophrenia; 27 applications resulting in an approval were therefore retained for further analysis. Their release dates ranged between 1996 and 2016 (median = 2008). One of these was a second application for agomelatine (orally) for major depressive disorder. The applications concerned 13 drugs, among which aripiprazole, olanzapine, paliperidone and duloxetine have been the subject of numerous authorisation applications. Sixteen applications concerned the oral route, eight concerned the intra-muscular route and three concerned other routes (sublingual and inhalation). More details, including the manufacturers' descriptions, are provided in Table 1.

**Table 1.** Details of the different evaluations leading to approval

EPAR	Year	Drug	Route	Disease	Manufacturer
EMEA/H/C/000916	2008	Agomelatine	O	Depressive disorders	Servier
^a^	2004	Aripiprazole	O	Schizophrenia	Otsuka
EMEA/H/C/000471/II/0015	2006	Aripiprazole	IM	Agitation in patients with schizophrenia and/or bipolar disorders	Otsuka
EMEA/H/C/471/II/0039	2008	Aripiprazole	O	Bipolar disorders	Otsuka
EMEA/H/C/471/II/0041	2008	Aripiprazole	IM	Agitation in patients with schizophrenia and/or bipolar disorders	Otsuka
EMEA/H/C/002755/0000	2013	Aripiprazole	IM	Schizophrenia	Otsuka
EMEA/H/C/001177	2010	Asenapine	Sublingual	Bipolar disorders	Organon
^a^	2006	Buprenorphine/naloxone	Sublingual	Addictive disorders	Schering-Plough Europe
^a^	2004	Duloxetine	O	Depressive disorders	Eli Lilly Nederland BV
EMEA/H/C/572/II/27	2008	Duloxetine	O	Anxiety disorders	Eli Lilly Nederland BV
EMEA/H/C/572/II/0036	2009	Duloxetine	O	Depressive disorders	Eli Lilly Nederland BV
EMEA/H/C/002400	2012	Loxapine	INH	Agitation in patients with schizophrenia and/or bipolar disorders	AlexzaUk
EMEA/H/C/002713/0000	2014	Lurasidone	O	Schizophrenia	Takeda Pharma A/S
EMEA/H/C/002583/0000	2012	Nalmefene	O	Addictive disorders	Lundbeck
EMEA/H/C/000890	2008	Olanzapine	IM	Schizophrenia	Eli Lilly Nederland BV
CPMP/0646/96	1996	Olanzapine	O	Schizophrenia	Eli Lilly Nederland BV
CPMP/0646/96	2003	Olanzapine	O	Bipolar disorders	Eli Lilly Nederland BV
CPMP/0646/96	2001	Olanzapine	IM	Agitation in patients with schizophrenia and/or bipolar disorders	Eli Lilly Nederland BV
CPMP/0646/96	2002	Olanzapine	IM	Agitation in patients with schizophrenia and/or bipolar disorders	Eli Lilly Nederland BV
^a^	2007	Paliperidone	O	Schizophrenia	Janssen-Cilag
EMEA/H/C/000746/II/0023	2010	Paliperidone	O	Schizoaffective disorder	Janssen-Cilag
EMEA/H/C/000746/II/0043	2015	Paliperidone	O	Schizoaffective disorder	Janssen-Cilag
EMEA/H/C/004066/X/0007/G	2016	Paliperidone palmitate long acting injection	IM	Schizophrenia	Janssen-Cilag
EMEA/H/C/2105	2011	Paliperidone palmitate long acting injection	IM	Schizophrenia	Janssen-Cilag
EMEA/H/C/000546/II/0004	2006	Pregabalin	O	Anxiety disorders	Pfizer
^a^	2006	Varenicline	O	Addictive disorders	Pfizer
EMEA/H/C/002717	2013	Vortioxetine	O	Depressive disorders	Lundbeck

EPAR, European Public Assessment Report; O, oral route; IM, intramuscular; INH, inhalation.

a These EPARs had no identifying number on EMA's website.

### Description of the EPARs

#### Evidence in trials *v*. active comparator

**Fig. 1.**
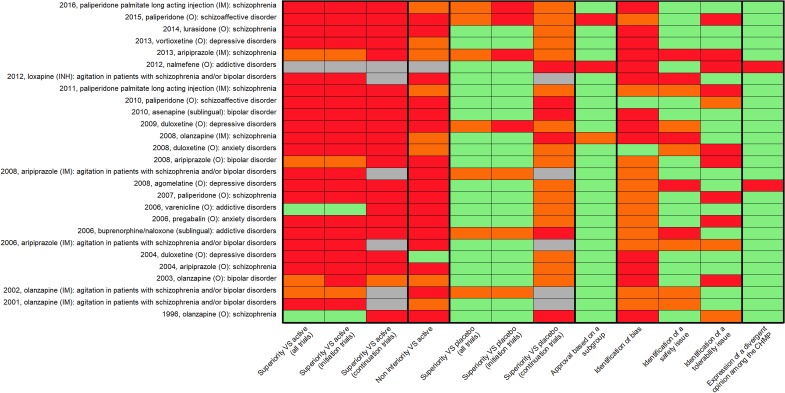
Heatmap presenting a descriptive analysis of the evaluations leading to approval. Columns 1–7: green = 2 or more studies; orange = one study; red = no study; column 8: green = not based on subgroup analyses; red = based on *a posteriori* subgroup analyses; column 9: green = no bias was identified; orange = bias assessment not presented; red = identification of bias; columns 10–11: green = no issue was identified; orange = issue presented as possible; red = identification of an issue; column 12: green = no divergent opinion; red = divergent opinion.

A description of all the EPARs is summarised in Fig. 1. For one evaluation (nalmefene, oral – (O), in alcohol use disorders), the comparative effectiveness was explicitly considered as not needed by the CHMP. Among the remaining approvals, 2/26 (8%) applications leading to approval were based on at least two initiation trials showing superiority against active comparator (olanzapine (O), for schizophrenia and varenicline (O), for smoking cessation), and 3/26 (12%) were based on one positive superiority study against active comparator. In total, 21/26 (80%) did not present any evidence of superiority against an active comparator: for 9 (35%) an active comparator arm was presented without any comparison with the investigation drug (this was described in the EPARs as ‘internal positive control for assay sensitivity’), for 9 (35%) no superiority study including an active comparator was presented, and for 3 (11%) superiority trials against active comparator presented a negative (i.e. non-significant) result.

In total, 1/26 (4%) of the applications gaining approval (duloxetine (O), for major depressive episode) provided evidence of two positive non-inferiority analyses *v*. active comparator. However, these two analyses were based on pooled analyses of different superiority trials. In total, 8/26 (31%) approved applications providing evidence of one positive non-inferiority study against active comparator, including one with one positive trial but two negative trials (paliperidone (IM), in maintenance treatment of schizophrenia) and another one based on a pooled analysis of two non-inferiority trials (duloxetine (O), for generalised anxiety disorder). In total, 17/26 (65%) evaluations provided no non-inferiority study against active comparator.

We considered that evidence from continuation trials was not needed in the five evaluations concerning agitation. Only one of the remaining 21 approvals (5%) provided evidence of non-inferiority against active comparator. It was based on only one study. None provided evidence of superiority against active comparator in continuation trials. In total, 2/21 (9.5%) reported one negative continuation trial *v*. active comparator. Because it was sometimes arbitrary to differentiate continuation and initiation trials (e.g. for long-acting injectable treatments the initiation of the IM route often follows stabilisation with the same drug given orally), we decided to group these two categories for descriptive purpose (Fig. 1). Details of these approvals in terms of study design and comparators explored are presented in Table 2. Details of outcomes presented in these approvals are presented in online appendix, e-Tables 1a and 1b.

**Table 2.** Approvals with evidence of superiority or non-inferiority against active comparator

Evaluation	Study: design (primary outcome/arms)	Comparator	Study duration^a^	Number of subjects
Positive superiority initiation trials *v*. active comparator				
Aripiprazole (O): bipolar disorders	CN138008: response/aripiprazole flexible dose 15–30 mg/day and comparator	Haloperidol, flexible dose, 10–15 mg/day	12 weeks	347 (randomised)
Olanzapine (O): schizophrenia	FID-EW-E003: not specified/olanzapine fixed doses 1–17.5 mg/day and comparator	Haloperidol, fixed doses, 10–20 mg/day	6 weeks	431 (initial)
Olanzapine (O): schizophrenia	FID-MC-HGAJ: not specified/olanzapine 5–20 mg/day and comparator	Haloperidol, 5–20 mg/day	6 weeks	1996 (included)
Olanzapine (IM): agitation in manic episode	HGHW: PANSS excited component/olanzapine fixed dose 10 mg, placebo and comparator	Lorazepam IM, fixed dose, 2 mg	2 h	228 (screened)
Varenicline (O): smoking cessation	A3051028: 4 weeks continuous quit rate/varenicline, fixed dose, 2 mg/day, placebo and comparator	Bupropion, fixed dose, 300 mg/day	12 weeks	1025 (randomised)
Varenicline (O): smoking cessation	A3051036: 4 weeks continuous quit rate/varenicline, fixed dose, 2 mg/day, placebo and comparator	Bupropion, fixed dose, 300 mg/day	12 weeks	1027 (randomised)
Positive non-inferiority continuation trials *v*. active comparator				
Olanzapine (O): bipolar disorder	HGHT: relapse/olanzapine and comparator. NIB: 7.3%	Lithium	12 months	431 (randomised)
Positive non-inferiority initiation trials *v*. active comparator				
Aripiprazole (IM): schizophrenia	31-07-247: relapse/aripiprazole IM depot flexible dose 300–400 mg/month, aripiprazole IM depot flexible dose 25–50 mg/month and comparator. NIB : 11.5%	Oral aripiprazole flexible dose 10–30 mg/day	26 weeks	662 (randomised)
Duloxetine (O): depressive disorder (pooled results)	Pooled F1J-MC-HMAT(a,b): HAMD17/duloxetine fixed doses 40 and 80/day, placebo and comparator. NIB: 2.2	Paroxetine fixed dose 20 mg	8 weeks	707 (randomised)
Duloxetine (O): depressive disorder (pooled results)	Pooled F1J-MC-HMAY(a,b): HAMD17/duloxetine fixed doses 80 and 120 mg/day, placebo and comparator. NIB: 2.2	Paroxetine fixed dose 20 mg	8 weeks	759 (randomised)
Duloxetine (O): anxiety disorders (pooled results)	Pooled HMDU, HMDW: HAMA/duloxetine flexible dose 60–120 mg/day, placebo and comparator. NIB: −1.5	Venlafaxine flexible dose 75–225 mg/day	10 weeks	1068 (randomised)
Olanzapine (IM): schizophrenia	F1D-MC-HGKA: relapse/olanzapine IM depot fixed dose 150 and 300 mg/2 weeks and comparator. NIB: 5%	Oral olanzapine fixed dose 10–20 mg/day	24 weeks	1065 (randomised)
Olanzapine (O): bipolar disorder	HGHQ: Y-MRS/olanzapine 5–20 mg/day and comparator	Valproate 500–2500 mg/day	3 weeks	251 (randomised)
Olanzapine (IM): agitation (in schizophrenia)	F1D-MC-HGHB: PANSS excitement component/olanzapine fixed dose, placebo and comparator	Haloperidol IM	2 h	311 (type of population not given)
Paliperidone (IM): schizophrenia (3-month injection)	PSY-3011: relapse/paliperidone IM depot fixed doses 175–525/12 weeks and comparator. NIB: 15%	Paliperidone fixed dose 50–150 mg/month	48 weeks	1016 (randomised)
Paliperidone (IM): schizophrenia	PSY-3006: PANSS/paliperidone IM depot flexible dose 50–150 mg/month and comparator. NIB: 5	Risperidone IM flexible dose 25–50 mg/2 weeks	13 weeks	1220 (randomised)
Vortioxetine (O): depressive disorders	14178A: MADRS/vortioxetine flexible dose 10–0 mg/day and comparator. NIB: 2	Agomelatine flexible dose 25–50 mg/day	12 weeks	501 (randomised)

NIB, non-inferiority boundary.

Missing data (for doses, route, NIB) in the table are due to missing data in the EPARs.

a Duration between randomisation and endpoint.

Quantitative information on the comparison of safety outcomes with an active comparator was presented for 17/26 (65%). In addition, for 2/26 (8%) evaluations the comparison was described qualitatively. A comparison of tolerance outcomes with active comparator was provided for 22/26 (85%) evaluations (21 quantitatively and 1 qualitatively).

#### Evidence *v*. placebo

In total, 20/27 (74%) evaluations resulting in approval reported evidence of superiority *v*. placebo with two or more initiation trials. In 3/27 (11%) superiority *v*. placebo was based on one initiation trial. In total, 16/22 (73%) evaluations reported evidence from one positive continuation study against placebo (two of these also reported failed trials) and six did not report evidence of such studies.

We also decided to group these two categories for descriptive purpose (Fig. 1) resulting in 20/27 evaluations resulting in approval with evidence of superiority *v*. placebo with two or more trials and seven with a single positive trial.

Quantitative information on the comparison of safety outcomes *v*. placebo was presented for 21/27 (78%) evaluations. In addition, for 2/27 (7%) evaluations the comparison was described qualitatively.

A comparison of tolerance outcomes *v*. placebo was provided for 26/27 (96%) evaluations (25 quantitatively and 1 qualitatively).

#### Other characteristics of the evidence presented in the EPARs

All other characteristics of the evidence presented in the EPARs are detailed in the online appendix (e-results, e-Tables 2, 3 and 4).

### Description of the studies included in EPARs for approved medications

#### Missing information

We identified 137 eligible RCTs in EPARs for approved medications. Data needed to determine effect sizes were fully available for these studies in seven EPARs, corresponding to 41 studies. Complete data were lacking for 18 EPARs, where 24 studies were adequately reported and 89 lacked sufficient data for direct calculation of effect size. All the study reports of these 89 studies were requested from the EMA via its website. All the receipt confirmations had been obtained on 3rd July 2017. We stopped data collection 1 year later, on 21st July 2018. At that time, three other EPARs had been completed, which corresponded to 19 additional studies. We also used the data concerning nalmefene obtained for previous work by of one of the present authors (Palpacuer *et al*., 2015), corresponding to five additional studies.

**Fig. 2.**
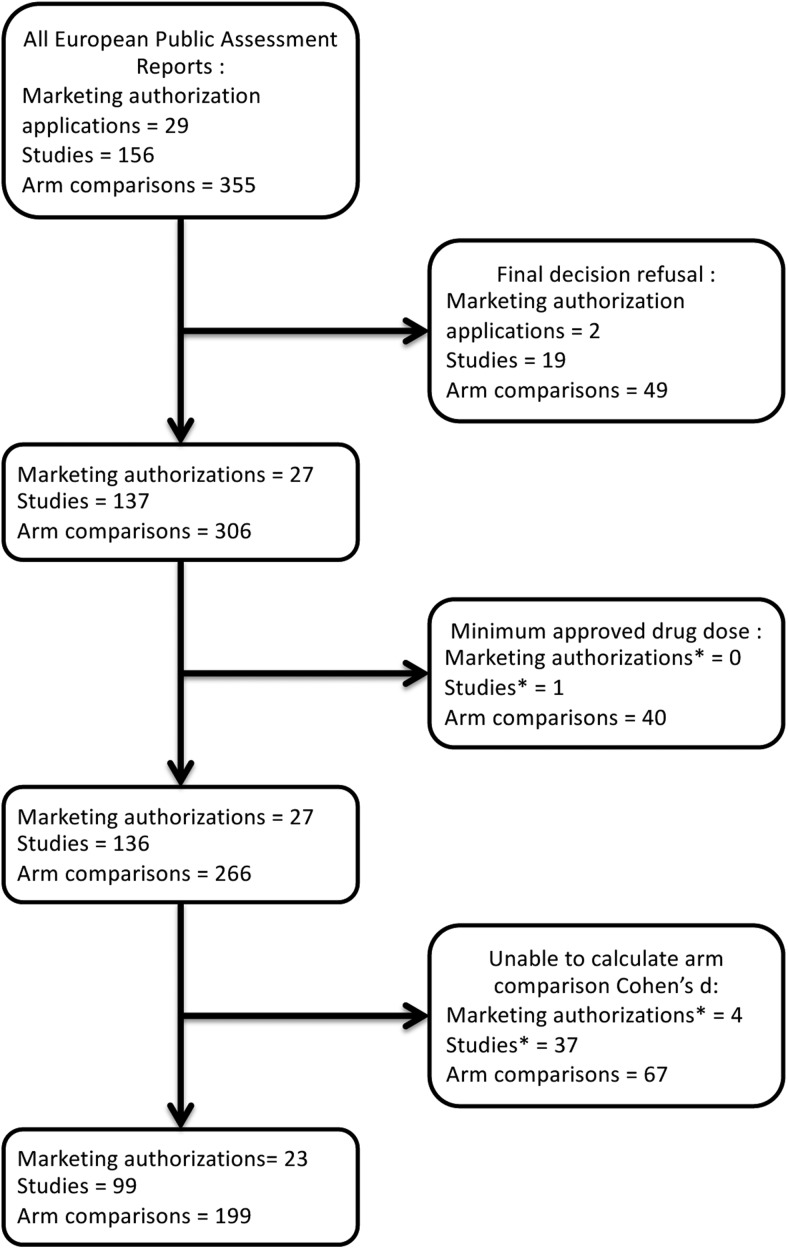
Flowchart of marketing authorisation application, individual studies and arm comparisons, for EPARs on psychiatric drugs. *Study/marketing authorisation totally excluded because all of its arm comparisons were excluded.

Effect size calculations and meta-analyses were performed only for approved dosages for approved medications. For one EPAR-approved study (Olanzapine (O), in schizophrenia), all dosages presented were below the minimum approved dosage, and the study was not included in meta-analyses. Therefore, we analysed 266 arm comparisons between the drug tested and placebo or active comparator in 136 studies. For these comparisons, 158/266 accounting for 78 studies (57%) presented sufficient information to calculate effect sizes and *p* values from arm results (proportions or mean changes and variance of mean changes), 29 enabled calculation of differences in arm results, adding five studies (4%) and 12 only presented *p* values and numbers of subjects, adding seven more studies (5%). Finally, 67/266 (25%) comparisons in 46 studies (34%) had insufficient information to calculate Cohen's *d*. For 37 studies (26%), none of the arm comparisons had sufficient information to calculate Cohen's *d*. After imputation of *p* values and numbers of subjects, we were able to calculate the effect sizes for at least one study in 23 (85%) EPARs, and for all studies in 12 (48%) EPARs. Results sufficient for meta-analysis concerned 199 comparisons (75%) in 99 studies (73%) (see the flowchart presented in Fig. 2).

**Fig. 3.**
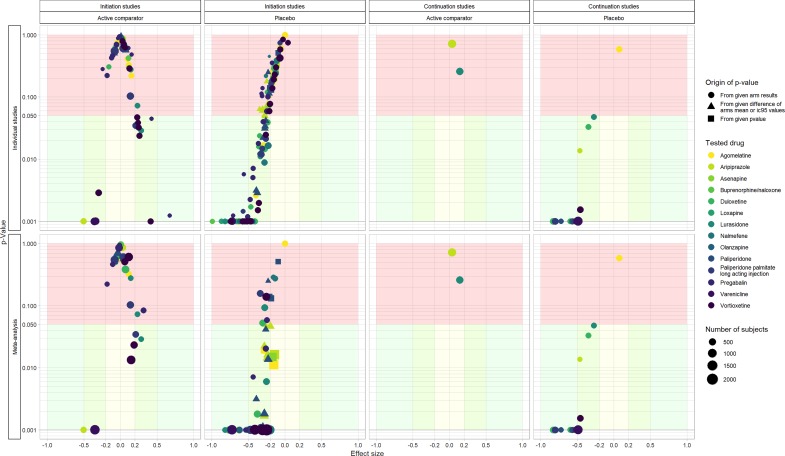
Effect sizes and *p* values observed in individual studies and meta-analyses pooled by drug and daily dose, for each study design. For each plot, the *x*-axis presents effect sizes and the *y*-axis (log scale) presents *p* values. Top plots present data at the study level and bottom plots present data at the meta-analysis level (data were pooled by drug and daily dose).

The relationship between effect-size and *p* values in the studies included and the results of the meta-analyses are presented in Fig. 3. Details per drug class are provided in online e-table 5.

#### Effect sizes in initiation studies against active comparator

For initiation studies against active comparator, we describe 57 different comparisons. Of these 13 (23%) results were considered statistically significant, 4 (7%) were in favour of the investigation drug and 9 (16%) were in favour of the active comparator. These comparisons involved 31 different combinations of drug and daily dose, 16 of which were explored only once. When results were pooled (meta-analyses) per drug and daily dose, we found 25 (81%) drug/dose comparisons with no evidence of superiority *v*. active comparator, 2 (7%) drug/dose comparisons with results in favour of the investigation drug and 4 (13%) drug/dose comparisons with results in favour of the active comparator. In these meta-analyses, the median effect size was 0.051 (range −0.503; 0.318).

#### Effect sizes in initiation studies against placebo

For initiation studies against placebo, we describe 125 different comparisons for 54 different combinations of drug and daily dose, 16 explored only once. Of these, 66 (53%) results were considered statistically significant. When results were pooled (meta-analyses) per drug and dose, we found 12 (23%) drug/dose comparisons with no evidence of superiority and 41 (77%) drug/dose comparisons with results in favour of the investigation drug. In these meta-analyses, the median effect size *v*. placebo was −0.283 (range −0.820; 0.091).

#### Effect sizes in continuation studies against active comparator

For continuation studies against active comparator, we describe two different comparisons. No results were considered statistically significant. These two comparisons concerned two different drugs. The effect sizes were 0.036 and 0.143.

#### Effect sizes in continuation studies against placebo

For continuation studies against placebo, we describe 15 different comparisons. Of these 14 (93%) results were considered statistically significant. Each combination of drug and daily dose was noted only once. In these comparisons, the median effect size was −0.528 (range −0.831; 0.074).

#### Exclusion of suicidal participants

Finally, in 48/137 studies (35%) we noted that suicidal participants were not included. For the other studies, no information was given concerning inclusion or not of suicidal participants.

## Discussion

### Statement of principal findings

Our critical appraisal of 25 EPARS revealed that EMA's standards for psychiatric drug approvals were low, especially for the consideration of comparative effectiveness issues. Only two applications receiving approval were based on at least two initiation trials showing superiority against active comparator (8%) and two others were based on only one study (8%). In one case, nalmefene (O), for reduction of alcohol consumption, the applicant considered that comparative effectiveness results were not needed, as the therapeutic goal was new, consisting of reducing alcohol consumption rather than maintaining alcohol abstinence. This has proved to be controversial, as nalmefene is a very similar compound to naltrexone, a drug already used off-label to reduce alcohol consumption (Naudet *et al*., 2016).

Interestingly, some EPARs presented studies with an active comparator but did not report the results of the comparison, and the CHMP did not consider this evidence in the decision process. In these cases, the active comparator was only compared to the placebo control to demonstrate ‘the sensitivity of the trial’. In other words, if the active comparator showed no difference *v*. placebo, the trial was considered as ‘a failed trial’, thus disqualifying any absence of difference observed with the drug under investigation. This is a serious issue as it is insufficient to consider a trial as ‘failed’ when an active comparator was not significantly better than placebo, especially given the large number of ‘negative’ trials of this sort (Turner *et al*., 2008).

Conversely, in certain non-inferiority studies such as study 14178A, comparing vortioxetine with agomelatine in major depressive episode, one would have expected a placebo arm to make it clear whether the two drugs were equally effective or equally ineffective. This example is of concern because agomelatine could be considered as a very poor comparator, since its approval was controversial: it was granted after a second application (the first application was refused) with a mention of divergent opinions among the CHMP members (Barbui and Cipriani, 2012; Koesters *et al*., 2013). While in the recent network meta-analysis by Cipriani *et al*. (2018), agomelatine ranked first if one considered the ratio between efficacy and tolerability, another meta-analysis suggested ‘that a clinically important difference between agomelatine and placebo in patients with unipolar major depression is unlikely’ (Koesters *et al*., 2013).

In fact, most of the approvals were solely based on evidence of superiority against placebo, which appears to be the standard for EMA. As a consequence, our meta-analyses revealed that in most of the cases, there was no added benefit with the most recent drugs and some approved doses for some drugs were less effective than those of already marketed drugs. It can be noted that these differences can be considered as small or very small in terms of effect size.

In addition, almost half of the comparisons *v*. placebo we considered did not reach statistical significance, a result observed in the antidepressant trials submitted to the FDA (Turner *et al*., 2008), suggesting that most of the pivotal trials lack power given the actual efficacy of the drug studied. Importantly, some approved doses for some drugs did not demonstrate effectiveness against placebo in our meta-analyses. When compared with placebo, the median effect size was small for all the doses of each drug explored. Larger effect sizes were observed in continuation studies, a design that is known to inflate the true treatment effect (Kopec *et al*., 1993), possibly in part because of a withdrawal effect and/or a selection of enriched sample of drug responders.

Further to this, while some evaluations identified safety issues that were general and related to drug classes (e.g. cardiovascular risks for antipsychotics or suicide risk for antidepressants) other evaluations reported issues that were specific to a given drug. For instance, long-acting IM depot olanzapine, besides the classic metabolic risks, presents a risk of inadvertent intravascular (IAIV) injection, resulting in a reduced level of consciousness, and coma in the worst cases. As the approval is based on a non-inferiority trial against oral olanzapine, it seems that the only identified difference between these two treatments was the IAIV injection risk. Other specific risk domains were liver function for agomelatine (a drug with poor evidence of effectiveness) and bronchospasm for inhaled loxapine. Inhaled loxapine was only compared to placebo while the oral and IM routes for loxapine were long available without this specific safety issue.

While in most EPARs the definition of the target population was not based on subgroup analyses, two evaluations used *a posteriori* subgroup analyses to define the target population: nalmefene (O) for reduction of alcohol consumption and paliperidone (O) for the depressive symptom domain of schizoaffective disorder. These analyses could increase the probability of false-positive findings (Wallach *et al*., 2017) and have no place in defining a target population.

### Strengths and weaknesses of the study

The criteria we used could be considered as controversial. For instance, the criterion of ‘at least two positive studies’ is classic, but it has been criticised, as it can still cover a weak level of evidence (van Ravenzwaaij and Ioannidis, 2017). It can be considered as too restrictive, since there are other benefits of new approved drugs (such as better observance, or better tolerance). Nevertheless, we extracted every primary outcome in every RCT and we found no trial exploring and demonstrating this kind of benefit. Primary outcomes are most of the time related to symptom severity, in a quantitative manner (symptom severity scale) or in a binary manner (relapse, response), depending on the study design. In addition, other outcomes like global functioning or quality of life, if present, are systematically assessed as secondary outcomes. In addition, because our aim was to assess the broad picture of drug approvals in psychiatry, we focused on very broad criteria and did not retain more qualitative outcomes involving the subtleties of day to day therapeutic practice. For example, we did not collect inclusion criteria for different studies and we were not able to discuss the representativeness of the samples included (Zimmerman *et al*., 2005; Naudet *et al*., 2013).

Data extraction in different EPARs proved to be difficult in certain situations, due to a lack of standardisation among EPARs. This was all the more so for adverse events that could be described quantitatively or qualitatively. In addition, EPARs summarise all the evidence analysed for granting marketing approval but they do not report the exact content of the market approval meetings. In addition, gathering data proved to be difficult, since details of studies were inconsistently reported in the EPARs (Barbui *et al*., 2011) and requests for study reports resulted in a long and still ongoing process, despite EMA's commitment to transparency. Our analyses therefore give an incomplete picture of the evidence base for these approvals. The next steps in our project are to finish data collection and extraction, to share an online interactive tool monitoring EMA's approvals of psychotropic medication and to update our database with the most recent approvals. Following the spirit of living systematic reviews (Elliott *et al*., 2017), this tool will be regularly updated, as we are operating in a fast-changing environment. For instance, since our searches, brexpiprazole and cariprazine have been approved for schizophrenia and esketamine for depression. Interestingly, the approval of esketamine has proved controversial (Cristea and Naudet, 2019, Gastaldon *et al*., 2019) suggesting the need for a continuous assessment of this type.

We could only assess published EPARs and found only two evaluations resulting in a refusal: one for agomelatine (the drug was approved later) and one for asenapine in schizophrenia (it was approved for bipolar disorder in the same EPAR). We had no access to unpublished and/or refused applications. Butlen-Ducuing *et al*. had access to applications of this type and found 46 applications in psychiatry between 1995 and 2014 (Butlen-Ducuing *et al*., 2016). We were unable to compare the applications that resulted in an approval with refused applications. In addition, we did not systematically appraise the EMA guidelines that are available online (EMA, 2018*a*). This is currently being done by another ongoing undertaking (Boesen *et al*., 2020), and it will be interesting to confront findings of this study with ours to explore whether EMA's guidelines were followed or not.

Finally, our study is not exhaustive for all psychotropic drugs approved in Europe, as these drugs can be authorised via other procedures (e.g. national procedure and decentralised/mutual recognition). And indeed, the situation may be even worse, as suggested by the example of reboxetine, an antidepressant that was approved for marketing in many European countries (e.g. the United Kingdom and Germany) in 1997 while it was an ineffective and potentially harmful antidepressant (Eyding *et al*., 2010). More recently, baclofen was approved by the French national agency for medicine and health product safety (the Agence Nationale de Sécurité du Médicament et des Produits de Santé [ANSM]), despite the evidence-based assessment by its Temporary Special Scientific Committee of independent experts who concluded that the risk–benefit ratio of baclofen in alcohol use disorder was negative (Naudet and Braillon, 2018).

## Conclusions

The evidence for psychiatric drug approvals by the EMA is in general low, especially when comparative effectiveness issues are considered. This result compounds many others that raise criticisms of EMA's criteria, not only in the field of psychopharmacology (Barbui *et al*., 2011; Banzi *et al*., 2014*a*, *b*). In addition, despite its commitment to sharing study report data, retrieval of these data proved to be difficult, combining a slow process of retrieval along with the concealment of EPARs reporting non-approval. These doubtful approvals and the lack of transparency could incentivise ineffective drug development and waste research efforts (Chalmers and Glasziou, 2009; Glasziou *et al*., 2014; Ioannidis *et al*., 2014). Output from independent and continuous monitoring by health authorities (such as the EMA) should motivate the definition of high standards, so as to drive the research agenda and avoid wasted efforts, and to reduce persistent uncertainties. Simple solutions can be proposed. First, EPARs should include proper meta-analyses (Chalmers and Glasziou, 2009; Glasziou *et al*., 2014), with data sharing of all aggregated data in a structured form allowing a complete overview of the evidence base for approved drugs. Ideally, to provide complete transparency, these meta-analyses should be prospective (prior to the RCTs). In the spirit of Open Science, we have suggested a model of ‘registered drug approvals’ following the data-blind peer-review model of registered reports (Naudet and Cristea, 2020). With suitable success criteria in place, this model would guarantee transparency in establishing the evidence required for approving a new drug.

## Supplementary material

For supplementary material accompanying this paper visit http://dx.doi.org/10.1017/S2045796020000359. click here to view supplementary material

## Data

The R session information, code and data used are available on Open Science Framework at https://osf.io/j9c26, DOI 10.17605/OSF.IO/J9C26.
