# Supplementary material for: The evidence base for psychotropic drugs approved by the European Medicines Agency: a meta-assessment of all European Public Assessment Reports
Source: Epidemiol Psychiatr Sci. 2020 Apr 27;29:e120. doi: 10.1017/S2045796020000359 (PMC7214735; doi:10.1017/S2045796020000359)
Supplement: Supplementary file 1 [file S2045796020000359sup001.docx]

**e-methods: Formulas applied to calculate the effect sizes**

For binary outcomes, Cohen’s d values (and variance) were derived from odds ratios with formulas (1) and (2):

(1) and,

(2)where V stands for variance ^12^.

For quantitatives outcomes, Cohen’s d values (and their variance) were derived from raw means and standard deviations with formulas (3), (4) and (5) :

(3) $d=\frac{\left( {Mean}_{1}-{Mean}_{2} \right)}{S_{pooled}}$ ,

(4) $S_{pooled}=\sqrt{\frac{\left( n_{1}-1 \right)S_{1}^{2}+\left( n_{2}-1 \right)S_{2}^{2}}{\left( n_{1}+n_{2}-2 \right)}}$ where n and S^2^ stand for the number of subjects and the variance for each group.

(5) $V_{d}=\frac{n_{1}+n_{2}}{n_{1}*n_{2}}+\frac{d^{2}}{2\left( n_{1}+n_{2} \right)}$

When only partial results where available, pooled variance and differences in means were calculated from the 95% CI with formula (6):

(6) $S_{pooled}=\frac{\frac{{95\text{\%}IC}_{h}-{95\text{\%}IC}_{l}}{2}}{\frac{1.96}{\sqrt{n_{total}}}}$ where 95% IC_h_ and IC_l_ stand for high and low boundaries of 95% IC and n_total_ is the total number of subjects.

When only differences in means or individual means and numbers of subject were available, pooled variance was calculated from p-values using formula (7):

(7) $S_{pooled}=\frac{\left| {Mean}_{1}-{Mean}_{2} \right|}{\frac{Q_{n}\left( 1-pvalue \right)}{\sqrt{n_{total}}}}$ where Q_n_() stands for the quantile function for normal distribution. When the p value was approximate, we used the highest possible value, and 0.05 when results were qualified as significant without mention of a p-value.

**e-results: Other characteristics of the evidence presented in the EPARs**

15/27 (56%) evaluations reported an assessment of risk of bias in the studies included (without using any specific tool). A risk of bias was identified in 13 of these 15 evaluations (see **web appendix, e-Table 3** for a more detailed description).

5/27 (19 %) approved evaluations identified a safety issue and 6/27 (22%) identified a possible safety issue. As the distinction between safety and tolerability issues was somewhat arbitrary and may differ across all EPARs, details are presented in the **web-appendix, e-Table 4**, including the description of any adverse events reported in the EPARs.

9/27 (33%) evaluations leading to approval identified a tolerance issue and 3/27 (11%) identified a possible tolerance issue (details are presented in **web-appendix, e-Table 4**).

25/27 (93%) approvals were not based on subgroup analyses (among these, 17 presented subgroup analyses without using them to define the target population). 2/27 (7%) were based on *a posteriori* subgroup analyses (for paliperidone (O), in depressive expression of schizoaffective disorder and for nalmefene (O) in alcohol use disorder). Details of outcomes presented in these approvals are presented in **web appendix, e-Table 2**.

16/27 (59%) approvals were granted alongside a requirement for specific post-marketing studies. For 5/27 (19 %) evaluations, the CHMP asked another scientific group to decide (such as the Psychiatric Scientific Advisory Group, the Central Nervous System Scientific Advisory Group, the Bio-Statistics Working Group or an internal expert working group). Expression of divergent positions within the Committee for Medicinal Products for Human Use in the EMA were reported in 2 approvals (nalmefene (O), in alcohol use disorders and agomelatine (O), for major depressive episode). For nalmefene, the divergent opinion was that the treatment effect was modest, the target population was defined post-hoc, the number of withdrawals was high, the results of the various sensitivity analyses were inconsistent, and there was no direct evidence of harm reduction with the drug. For agomelatine, the divergent opinion stated that efficacy was not consistently demonstrated and the safety risk was unquantified.

The number of trials included in each approval is summarized in the **web appendix, e-Table 6**.

| **Primary outcome** | **Evaluations with evidence of superiority against active comparator** | **Evaluations with evidence of superiority against placebo** | **Evaluations with evidence of non-inferiority against active comparator** | **Evaluations with positive continuation studies against active comparator** | **Evaluations with positive continuation studies against placebo** |
| --- | --- | --- | --- | --- | --- |
|  | (n = 4) | (n = 24)* | (n = 9) | (n=1) | (n=16) |
| **Symptom severity scale (auto/hetero)** | 1 (25%) | 22 (92%) | 6 (67%) | 0 | 0 |
| **Clinical global impression (CGI)** | 0 | 0 | 0 | 0 | 0 |
| **Global functioning** | 0 | 0 | 0 | 0 | 0 |
| **Quality of life** | 0 | 0 | 0 | 0 | 0 |
| **Relapse** | 1 (25%) | 3 (12%) | 3 (33%) | 1 (100%) | 14 (87,5%) |
| **Response** | 1 (25%) | 0 | 0 | 0 | 0 |
| **Remission** | 0 | 0 | 0 | 0 | 0 |
| **Other** | 1 (25%) | 1 (4%) | 0 | 0 | 2 (12,5%) |

**e-Table 1a: Primary outcomes in evaluations with evidence of comparative effectiveness**

n: number of evaluations for each kind of evidence

* for 2 evaluations, 2 primary outcomes were used (a symptom severity scale and negative urine samples for bupernorphine/naloxone; a symptom severity scale and relapse for long-acting IM olanzapine)

| **All outcomes** | **Evaluations with superiority studies with an active comparator arm** | **Evaluations with superiority studies against placebo (initiation trials)** | **Evaluations with non-inferiority studies against active comparator** | **Evaluations with continuation studies against active comparator** | **Evaluations with continuation studies against placebo** |
| --- | --- | --- | --- | --- | --- |
|  | (n = 16) | (n = 24) | (n = 9) | (n = 3) | (n = 16) |
| **Symptom severity scale (auto/hetero)** | 16 (100%) | 24 (100%) | 9 (100%) | 2 (67%) | 11 (69%) |
| **Clinical global impressions (CGIs)** | 13 (81%) | 21 (87%) | 7 (78%) | 2 (67%) | 10 (62,5%) |
| **Global functioning** | 3 (19%) | 7 (29%) | 6 (67%) | 0 | 6 (37,5%) |
| **Quality of life** | 4 (25%) | 7 (29%) | 4 (44%) | 0 | 2 (12,5%) |
| **Relapse** | 3 (19%) | 5 (21%) | 3 (33%) | 3 (100%) | 14 (87,5%) |
| **Response** | 11 (69%) | 21 (87%) | 7 (78%) | 1 (33%) | 4 (25%) |
| **Remission** | 6 (37%) | 8 (33%) | 4 (44%) | 0 | 4 (25%) |
| **Other** | 8 (50%) | 15(62%) | 1 (11%) | 2 (67%) | 7 (44%) |

**e-Table 1b: All outcomes in all evaluations**

n: number of evaluations for each kind of study

| **Drugs (route) : indication based on subgroup analyses** | **Symptom severity scale (auto/hetero)** | **Clinical global impressions (CGIs)** | **Global functioning** | **Quality of life** | **Relapse** | **Response** | **Remission** | **Other** |
| --- | --- | --- | --- | --- | --- | --- | --- | --- |
| **Nalmefene (O): reduction of alcohol consumption in adult patients with alcohol dependence who have a high drinking risk l, (…) who continue to have a high drinking risk two weeks after initial assessment** | YES | YES | YES | NO | NO | YES | NO | alcohol dependance and abuse measures; biological laboratory test (e.g liver function); proportion of days where the treatment is taken |
| **Paliperidone (O): depressive symptom domain of schizoaffective disorder** | YES | YES | YES | NO | YES | NO | NO | medication satisfaction questionnaire; resource utilization questionnaire |

**e-Table 2: Details of outcomes for approvals based on a posteriori subgroup analyses**

| **Evaluation** | **Details (citations)** | **EPAR** |
| --- | --- | --- |
| **Aripiprazole (O) : schizophrenia** | The only trial that fulfils the recommended duration of follow-up has a quite high rate of dropout at 66%. | . |
| **Aripiprazole (IM) : schizophrenia** | The mean age (considered slightly higher than expected) and the long duration of oral stabilisation ( at least 8 weeks) may have been possible factors contributing to the lower than anticipated relapse rate observed in both studies. | EMEA/H/C/002755/0000 |
| **Asenapine (sublingual) : bipolardisorder** | The differences in the withdrawal rate presented a concern (as the withdrawals were mainly due to lack of efficacy). | EMEA/H/C/001177 |
| **Duloxetine (O) : depressivedisorders (MDE)** | Patients with severe MDD and elderly patients are clearly underrepresented in the available efficacy database. | . |
|  | The specified non-inferiority margin appears quite wide (2.2 in the HAMD17), and the reason for its selection is not considered justified by the CHMP. |  |
| **Duloxetine (O) : depressive disorders (MDD)** | Stringent response criteria were required to be eligible and the maintenance criteria used may limit the scope for extrapolating the results. | EMEA/H/C/572/II/0036 |
|  | ‘Recurrence’ and ‘re-emergence’ criteria are based on the investigator-based assessment (CGI-Severity) instead of a patient-based score (HAMD17), which would have been by far preferable. |  |
|  | The short duration of the trial (1-year maintenance treatment, when longer duration would have been preferable, closer to clinical practice and regulatory recommendations) does not enable an elucidation of any additional gain to be expected. |  |
| **Loxapine (INH) : agitation** | The CHMP was concerned about the exclusion criteria which may have limited the scope for generalizing the results. Patients apparently had moderate rather than severe agitation (cooperativeness, baseline PEC score). | EMEA/H/C/002400 |
| **Lurasidone (O) : schizophrenia** | The impact of dose adjustments in the early phase of the D1050234 study and the inclusion of a sub-therapeutic dose of quetiapine XR (200 mg) within a flexible dosing range | EMEA/H/C/002713/0000 |
|  | The duration of the initial trial may not have allowed for true stabilisation with potential carry-over effects from the previous short-term (6-week) study D1050233 to the extension and maintenance of effect in study D1050234, with an increased risk of discontinuation due to relapse during the initial phase of that study. | EMEA/H/C/002713/0000 |
| **Nalmefene (O) : addictive disorders** | The high withdrawal rate renders imputation of missing data an important issue when determining statistical significance of primary efficacy endpoints. There was an attempt to resolve this by sensitivity analyses applying various imputation methods. | EMEA/H/C/002583/0000 |
| **Olanzapine (IM) : schizophrenia** | Randomization is necessary but introduces a bias into the double-blind comparisons by creating an advantage for the randomly assigned oral olanzapine group, which remained on its previous dose before and after randomization. | EMEA/H/C/000890 |
| **Olanzapine (O) : schizophrenia** | Haloperidol doses were possibly too high and may have induced depression mimicking negative symptoms. | CPMP/0646/96 |
|  | The patient population enrolled was of the mixed type and it is difficult to affirm the efficacy of olanzapine on negative symptoms. |  |
| **Olanzapine (O) : bipolardisorder** | Heterogeneity of the patient population. Premature discontinuation following an interim analysis not planned in the protocol. | CPMP/0646/96 |
|  | Premature discontinuation due to an interim analysis not planned in the protocol. |  |
|  | Possibility of unblinding of investigators to treatment assignment |  |
| **Paliperidone (IM) : schizophrenia (3-month injection)** | It can be noted that nearly 60% of the study population included in the DB phase had had at least one hospitalization for psychosis within the last 24 months prior to the study start, while only 4% of subjects had psychiatric hospitalization during the DB phase | EMEA/H/C/004066/X/0007/G |
|  | It is considered that the PP3M treatment effect is mixed, with a certain unknown placebo effect due to the monthly placebo injections when active medication is not given. |  |
| **Vortioxetine (O) : depressivedisorders** | The exclusion of non-responders and the inclusion of previous responders in the active reference arm could have introduced a bias in favour of the efficacy of the active reference, and thus differences in the efficacy of Vortioxetine versus the active reference cannot be inferred on the basis of these studies. | EMEA/H/C/002717 |
|  | The CHMP did not agree with the definition of the patient population “patients with MDD who have responded inadequately to SRI antidepressant monotherapy”, for the following reasons: […] "It cannot be excluded that fully responsive patients to the first SRI monotherapy could have been included in the study since the severity of depression was not assessed at the onset of the first monotherapy during the lead-in period. |  |

**e-Table 3: Description of risk of bias identified in the different evaluations**

O: oral route; IM : intramuscular; INH : inhalation

. These EPARs had no identifying number on EMA’s website

| **Evaluation** | **Safety issue** | | **Tolerance issue** | | **Discontinuation rates due to adverse events** |
| --- | --- | --- | --- | --- | --- |
|  | **Identified** | **Possible** | **Identified** | **Possible** |  |
| **Agomelatine (O) :**  **depressive disorder** | Liver function | Suicide; Death of elderly patient with dementia | no | no | Not reported |
| **Aripiprazole (IM) :**  **agitation (schizophrenia)** | no | Association with parenteral benzodiazepines (cardiorespiratory depression) | no | Orthostatic hypotension | 0,7% (calculated) |
| **Aripiprazole (O) :**  **bipolar disorder** | no | no | Depression | no | Not reported |
| **Aripiprazole (IM) :**  **schizophrenia** | Low WBC values | no | Extra-pyramidal symptoms | no | Not reported |
| **Buprenorphine/ naloxone (sublingual) :**  **addictive disorders** | Spontaneous abortion and neonatal withdrawal syndrome | Patients with existing hepatitis and/or HCV infection are probably more likely to have greater increases in hepatic enzymes during treatment with buprenorphine than others without these complications. | no | no | 2.40% |
| **Duloxetine (O) :**  **anxiety disorders** | no | Suicide; Hepatic risk | Nausea and diarrhoea | no | 4,8 to 13,3% |
| **Duloxetine (O) :**  **depressive disorders** | no | Suicide | no | no | 3,7 to 17% |
| **Loxapine (INH) :**  **agitation (schizophrenia or bipolar disorder)** | Risk of severe broncospasm | Co-administration with other antipsychotics, in particular with regard to the potential increased risk of QT prolongation | no | no | 1,2% (for 004-201, 004-301, 004-302) |
| **Nalmefene (O) :**  **addictive disorders** | no | no | Nausea, dizziness, sleep-related events | no | 10,50% |
| **Olanzapine (IM) :**  **schizophrenia** | IAIV events (or post-injection syndrome) ; metabolic risks |  | no | no | Not reported |
| **Olanzapine (O) :**  **schizophrenia** | no | no | no | Weight gain | Not reported |
| **Olanzapine (O) :**  **bipolar disorder** | no | no | More adverse events compared to valproate ; Weight gain | no | Not reported |
| **Olanzapine (IM) :**  **agitation (schizophrenia)** | no | Cardiovascular safety | no | no | Not reported |
| **Olanzapine (IM) :**  **agitation (bipolar disorder)** | no | Cardiovascular safety | no | no | Not reported |
| **Paliperidone (O) :**  **schizophrenia** | no | no | Increase in BMI; Orthostatic hypotension; QT prolongation and arrhythmia | no | 5% (pooled double-blind safety analysis set) |
| **Paliperidone (O) :**  **schizo-affective disorder (psychotic or manic)** | no | no | no | Significant effect related to concomitant medication on the rate of ADRs was observed; Increased serum prolactin; Extra-pyramidal symptoms. | 7,00% |
| **Paliperidone (O) :**  **schizo-affective disorder (depressive)** | no | no | Extrapyramidal syndrome, hyperglycaemia, hyperprolactinaemia | no | 7,3% (for SCA-3004) |
| **Paliperidone (IM) :**  **schizophrenia** | no | Cardiovascular and cerebrovascular events in elderly patients | Local injection site reaction | no | 2 to 19,2% |
| **Pregabalin (O) :**  **anxiety disorders** | no | no | Oedema, weight gain | Discontinuation symptoms | 10,7% to 11,3% |

**e-Table 4: Details for evaluations with mention of safety and/or tolerance issues**

WBC : white blood cells; IAIV: inadvertent intravascular injection

| **Drug class** | **Number of comparisons** | **Number of comparisons with significant differences** | **Number of comparisons with significant differences in favor of the experimental drug** | **Median effect size at the study level (min-max)*** | **Number of unique comparisons**  **(i.e. number of meta-analyses)** | **Number of unique comparisons explored in only one study** | | **Number of meta-analyses with no significant results** | | **Number of meta-analyses with significant differences in favor of the experimental drug** | **Number of meta-analyses with significant differences in favor of the comparator** | **Median effect size at the meta-analysis level (min-max)*** |
| --- | --- | --- | --- | --- | --- | --- | --- | --- | --- | --- | --- | --- |
| **Initiation trials versus active** | | |  |  |  |  |  | |  |  | | |
| All | 57 | 13 (23 %) | 4 (7 %) | 0.044  (-0.503 to 0.676) | 31 | 16 (52 %) | | 25 (81 %) | | 2 (6 %) | 4 (13 %) | 0.051  (-0.503 to 0.318) |
| Antidepressant | 24 | 6 (25 %) | 1 (4 %) | 0.059  (-0.297 to 0.414) | 9 | 1 (11 %) | | 7 (78 %) | | 0 (0 %) | 2 (22 %) | 0.070  (-0.069 to 0.187) |
| Antipsychotic | 18 | 3 (17 %) | 1 (6 %) | 0.052  (-0.503 to 0.283) | 15 | 12 (80 %) | | 12 (80 %) | | 1 (7 %) | 2 (13 %) | 0.053  (-0.503 to 0.283) |
| Other | 15 | 4 (27 %) | 2 (13 %) | -0.054  (-0.357 to 0.676) | 7 | 3 (43 %) | | 6 (86 %) | | 1 (14 %) | 0 (0 %) | -0.018  (-0.346 to 0.318) |
| **Initiation trials versus placebo** | | |  |  |  |  |  | |  |  | | |
| All | 125 | 66 (53 %) | 66 (53 %) | -0.276  (-0.994 to 0.042) | 54 | 16 (30 %) | | 12 (22 %) | | 42 (78 %) | 0 (0 %) | -0.283  (-0.82 to 0) |
| Antidepressant | 42 | 23 (55 %) | 23 (55 %) | -0.260  (-0.741 to 0.042) | 13 | 3 (23 %) | | 3 (23 %) | | 10 (77 %) | 0 (0 %) | -0.305  (-0.518 to 0) |
| Antipsychotic | 56 | 28 (50 %) | 28 (50 %) | -0.274  (-0.869 to -0.067) | 29 | 10 (34 %) | | 6 (21 %) | | 23 (79 %) | 0 (0 %) | -0.281  (-0.82 to -0.091) |
| Other | 27 | 15 (56 %) | 15 (56 %) | -0.314  (-0.994 to -0.059) | 12 | 3 (25 %) | | 3 (25 %) | | 9 (75 %) | 0 (0 %) | -0.397  (-0.771 to -0.132) |
| **Continuation trials versus active** | | |  |  |  |  |  | |  |  | | |
| All | 2 | 0 (0 %) | 0 (0 %) | 0.089  (0.036 to 0.143) | 2 | 2 (100 %) | | 2 (100 %) | | 0 (0 %) | 0 (0 %) | 0.089  (0.036 to 0.143) |
| Antipsychotic | 2 | 0 (0 %) | 0 (0 %) | 0.089  (0.036 to 0.143) | 2 | 2 (100 %) | | 2 (100 %) | | 0 (0 %) | 0 (0 %) | 0.089  (0.036 to 0.143) |
| **Continuation trials versus placebo** | | |  |  |  |  |  | |  |  | | |
| All | 15 | 14 (93 %) | 14 (93 %) | -0.528  (-0.831 to 0.074) | 15 | 15 (100 %) | | 1 (7 %) | | 14 (93 %) | 0 (0 %) | -0.528  (-0.831 to 0.074) |
| Antidepressant | 6 | 5 (83 %) | 5 (83 %) | -0.506  (-0.831 to 0.074) | 6 | 6 (100 %) | | 1 (17 %) | | 5 (83 %) | 0 (0 %) | -0.506  (-0.831 to 0.074) |
| Antipsychotic | 7 | 7 (100 %) | 7 (100 %) | -0.569  (-0.804 to -0.273) | 7 | 7 (100 %) | | 0 (0 %) | | 7 (100 %) | 0 (0 %) | -0.569  (-0.804 to -0.273) |
| Other | 2 | 2 (100 %) | 2 (100 %) | -0.504  (-0.520 to -0.487) | 2 | 2 (100 %) | | 0 (0 %) | | 2 (100 %) | 0 (0 %) | -0.504  (-0.52 to -0.487) |

**e-Table 5: Effect estimates by drug class (at study and meta-analysis level)**

*: a negative difference indicates a comparison in favor of the experimental drug.

|  |  | **All studies** | **Initiation studies against Active comparator** | **Initiation studies against Placebo** | **Continuation studies against Active comparator** | **Continuation studies against Placebo** | **Unknown against Unknown** | **Unknown against Active comparator** |
| --- | --- | --- | --- | --- | --- | --- | --- | --- |
| All drugs | Approvals | 27 | 22 | 24 | 3 | 16 | 2 | 2 |
|  | Trials | 5 (2 - 8) | 2 (1.25 - 3) | 3.5 (2 - 6) | 1 (1 - 1) | 1 (1 - 1) | 1.5 (1.25 - 1.75) | 1 (1 - 1) |
| Antidepressants | Approvals | 5 | 4 | 4 | 0 | 5 | 1 | 1 |
|  | Trials | 7 (5 - 13) | 5 (3.5 - 6.25) | 6.5 (5.5 - 8.25) |  | 1 (1 - 1) | 1 (1 - 1) | 1 (1 - 1) |
| Antipsychotics | Approvals | 18 | 15 | 16 | 3 | 9 | 1 | 1 |
|  | Trials | 2.5 (2 - 7.25) | 2 (1 - 3) | 3 (2 - 4) | 1 (1 - 1) | 1 (1 - 1) | 2 (2 - 2) | 1 (1 - 1) |
| Other | Approvals | 4 | 3 | 4 | 0 | 2 | 0 | 0 |
|  | Trials | 7 (5.5 - 7.25) | 3 (2 - 4) | 6.5 (4.75 - 7) | NA | 1 (1 - 1) | NA | NA |

**e-Table 6: Number of trials in each approval**

Data are summarised using Median and Inter Quartile Range
